# Supplementary material for: Preparation, Physicochemical Properties, and In Vitro Toxicity towards Cancer Cells of Novel Types of Arsonoliposomes
Source: Pharmaceutics. 2020 Apr 6;12(4):327. doi: 10.3390/pharmaceutics12040327 (PMC7238025; doi:10.3390/pharmaceutics12040327)
Supplement: Supplementary file 1 [file pharmaceutics-12-00327-s001.pdf]

pharmaceutics

MDPI

Supplementary Materials: Preparation, Physicochemical Properties, and In Vitro Toxicity towards Cancer Cells of Novel Types of Arsonoliposomes

Paraskevi Zagana, Spyridon Mourtas, Anastasia Basta and Sophia G. Antimisiaris *

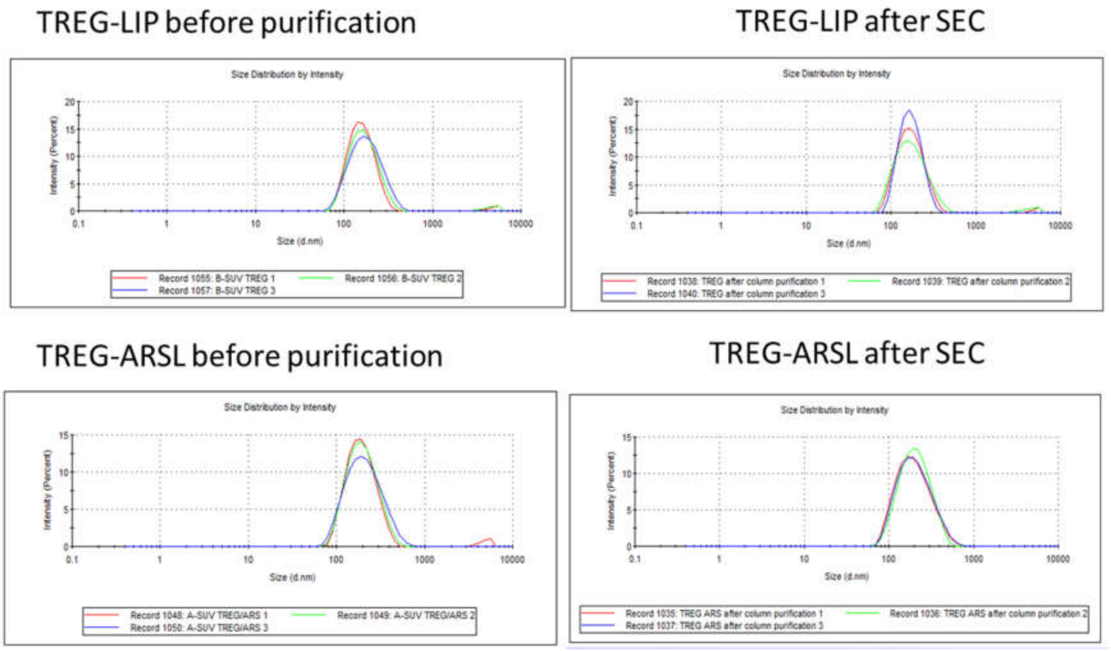

Figure S1. DLS size distribution graphs for TREG-LIP and TREG-ARSL, before and after purification by Size Exclusion Chromatography [SEC] (as reported in the Materials section).

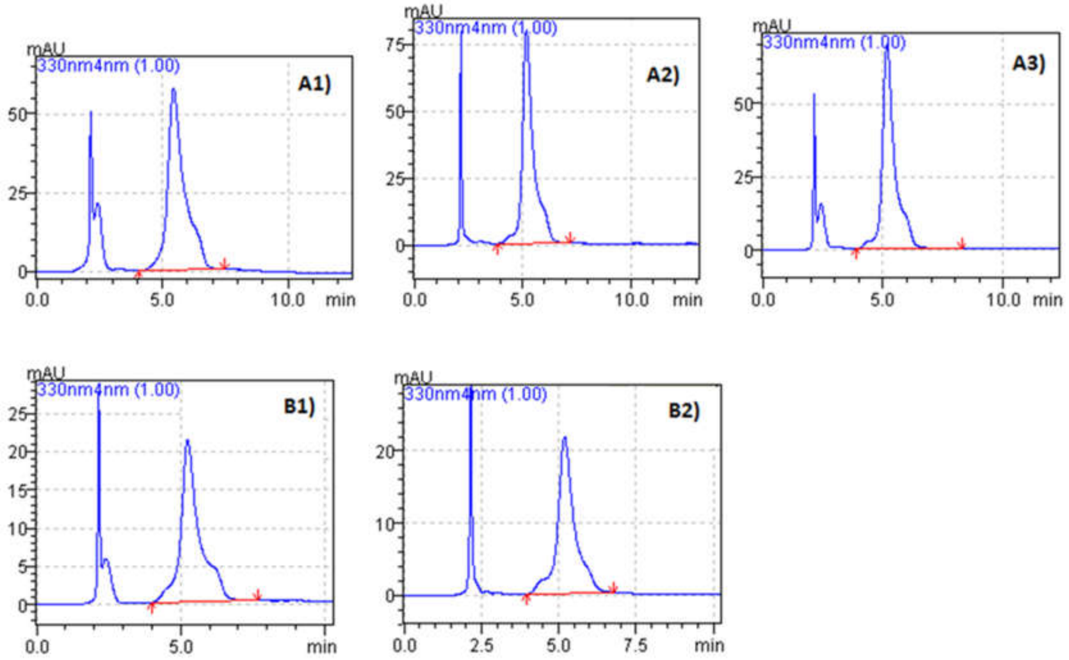

Figure S2. Representative chromatograms of Lipid-TREG after liposome disruption in 90% MeOH: Column: Lichrosphere 100 RP-18 LichroCART 250-4; flow rate: 1 ml/min, Mobile phase: 100% MeOH (isocratic); wavelength: 330nm. TREG-ARSL before purification (A1); after column purification (A2); after ultracentrifugation (A3). TREG LIP before purification (B1); and after column purification (B2).

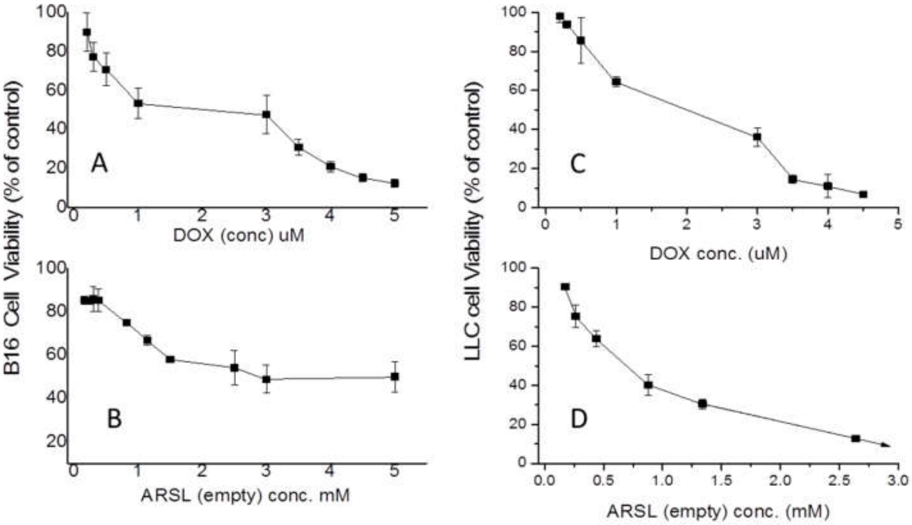

Figure S3. B16 and LLC cell viability after co-incubation with increasing concentrations of free DOX (solution) or empty ARSL. A. B16 cells /DOX; B. B16 cells/ARSL; C. LLC cells/DOX; D. LLC cells/ ARSL.
